# Vitamin D Status and Response to Calcifediol Supplementation in Term and Preterm Infants with Cholestasis

**DOI:** 10.3390/nu18182962

**Published:** 2026-09-09

**Authors:** Katarzyna Chada-Borowiecka, Justyna Czech-Kowalska, Edyta Czekuć-Kryśkiewicz, Ewa Skorupa, Marek Wójcik, Małgorzata Jarończyk, Rafał Bartczuk, Anna Niezgoda, Dariusz Gruszfeld

**Affiliations:** 1 Department of Neonatology and Neonatal Intensive Care, Children’s Memorial Health Institute, 04-730 Warsaw, Poland; k.chada@ipczd.pl (K.C.-B.);; 2 Biobank, Children’s Memorial Health Institute, 04-730 Warsaw, Poland; 3 Laboratory of Immunochemistry and Clinical Allergology, Children’s Memorial Health Institute, 04-730 Warsaw, Poland; 4 Pediatric Regional Center for Digital Medicine, Children’s Memorial Health Institute, 04-730 Warsaw, Poland

**Keywords:** vitamin D status, cholestasis, biliary atresia, calcifediol, preterm infants

## Abstract

Background/Objectives: Infants with cholestasis are at risk of vitamin D status impairment; the role of prematurity remains unclear. The primary aim of this study was to characterize vitamin D status in term and preterm infants with cholestasis and to determine whether serum 25OHD concentrations were associated with gestational age, etiology of cholestasis, and biochemical markers of cholestatic activity. The secondary aim was to assess changes in serum 25OHD concentrations during calcifediol supplementation and to identify factors associated with follow-up 25OHD concentrations. Methods: In this prospective observational study with a concurrent control group, infants with cholestasis and controls underwent assessment, including serum 25-hydroxyvitamin D (25OHD), at baseline and after calcifediol supplementation in cholestatic infants. Predictors of 25OHD were evaluated using multivariable models. Results: We included 127 infants: 70 with cholestasis (39 term, 31 preterm) and 57 controls (30 term, 27 preterm). Biliary atresia was diagnosed in 21 (30%) infants. Calcifediol was administered to 38 (54%) cholestatic infants: 18 (46%) term and 20 (64%) preterm at baseline. The remaining 32 infants with cholestasis were receiving vitamin D3 supplementation. Following enrollment and review of the baseline 25OHD results, calcifediol treatment was continued or initiated in 66 of the 70 infants. Treatment was discontinued in four infants based on their high serum 25OHD concentrations. Baseline 25OHD was similar in cholestasis and controls (34.81 ± 21.33 vs. 31.55 ± 10.14 ng/mL; *p* = 0.261). However, vitamin D deficiency (<20 ng/mL) was more frequent in cholestatic infants than controls (22.8% vs. 5%; *p* < 0.05), and 25OHD concentrations > 50 ng/mL were also more frequent (18.6% vs. 3.5%; *p* < 0.05). Among 13 infants with 25OHD concentrations > 50 ng/mL, 11 (84.6%) infants had received calcifediol; only three exceeded 100 ng/mL. Each 1 µg/kg increase in calcifediol dose was associated with 15.7% higher expected baseline 25OHD. At follow-up, preterm and term infants had comparable 25OHD (44.15 ± 20.36 vs. 45.73 ± 18.77 ng/mL; *p* = 0.737); nevertheless, <30 ng/mL persisted in 23%, while >50 ng/mL occurred in 40% of cholestatic infants. Baseline 25OHD concentration and cumulative calcifediol exposure were associated with follow-up 25OHD concentration. Conclusions: Vitamin D status in cholestatic infants is highly variable. Cumulative exposure of calcifediol is associated with higher 25OHD concentrations, but heterogeneous responses require monitoring and individualized dose adjustment.

## 1. Introduction

Cholestatic jaundice in infancy comprises a heterogeneous group of disorders characterized by impaired bile formation or bile flow, resulting in the accumulation of conjugated bilirubin and other biliary constituents in the liver and systemic circulation [1,2,3,4]. Cholestasis leads to profound disturbances in lipid digestion and absorption of lipids and fat-soluble vitamins. Consequently, the absorption and metabolism of vitamin D, as well as other fat-soluble vitamins (A, E, and K), may become impaired. Moreover, the liver plays a pivotal role in the hydroxylation and storage of vitamin D, further predisposing patients with cholestasis to vitamin D deficiency [1,2,3,5,6]. Vitamin D deficiency represents a particularly important clinical issue in this vulnerable population due to its essential role in calcium–phosphate homeostasis, skeletal mineralization, and bone health, as well as its increasingly recognized extraskeletal effects, including potential associations with prematurity-related morbidities such as respiratory distress syndrome and bronchopulmonary dysplasia [7,8,9]. Moreover, vitamin D deficiency has been linked to increased susceptibility to infections, including late-onset sepsis, as well as to hospitalizations for respiratory tract infections and gastroenterocolitis during the first year of life [10]. A growing body of evidence also suggests a role for vitamin D in vascular and developmental processes, as illustrated by the association between low 25OHD concentration and both the occurrence and severity of retinopathy of prematurity [11].

Recent evidence indicates that low 25OHD concentration may correlate with cholestasis severity and hepatic fibrosis in biliary atresia, underscoring the diagnostic and prognostic relevance of vitamin D status in cholestatic liver diseases [2,12]. Preterm infants represent a particularly vulnerable subgroup due to immaturity of the biliary system, limited third-trimester transfer of fat-soluble vitamins, rapid postnatal growth, increased renal losses, and frequent reliance on prolonged parenteral nutrition [1,13]. Preterm infants are at higher risk of vitamin D deficiency and may require higher supplementation doses than term infants (800 IU/day vs. 400 IU/day) to achieve adequate vitamin D status. However, to minimize the risk of hypo- and hypervitaminosis D, monitored and individualized supplementation appears to be the most appropriate approach [14,15].

Available data on vitamin D status in preterm infants with cholestasis remain limited and inconsistent, as most studies focus on term infants with biliary atresia [6,13,16]. Although current guidelines recommend routine monitoring and supplementation of fat-soluble vitamins in cholestatic infants, optimal dosing strategies, target serum levels, and safety thresholds—particularly for preterm infants—remain insufficiently defined [1,3,17]. Standard supplementation regimens designed for term infants may be inadequate or may increase the risk of overdosing in preterm infants due to altered pharmacokinetics and organ immaturity [18,19]. Existing vitamin D guidelines therefore emphasize individualized assessment in high-risk groups, including infants with liver disease and malabsorption syndromes [5,18,19].

The broader study protocol included an assessment of fat-soluble vitamins in infants with cholestasis; however, the present analysis focuses exclusively on vitamin D. Therefore, the primary aim of this study was to characterize vitamin D status in term and preterm infants with cholestasis and to determine whether serum 25OHD concentrations were associated with gestational age, cholestasis etiology, and biochemical markers of cholestatic activity. The secondary aim was to assess changes in serum 25OHD concentrations during calcifediol supplementation and to identify the factors associated with follow-up 25OHD concentrations. We hypothesize that preterm infants with cholestasis exhibit more pronounced vitamin D deficiency than term infants, that higher levels of biochemical markers of cholestatic activity would be associated with serum 25OHD concentration, and that standard supplementation regimens may be insufficient to ensure optimal vitamin D status in preterm infants with cholestasis.

## 2. Materials and Methods

### 2.1. Study Design and Setting

This prospective observational study with a concurrent control group was conducted at the Children’s Memorial Health Institute in Warsaw. Participant recruitment was carried out in the Department of Neonatology and Intensive Care of Newborns and Infants from December 2019 to December 2025.

The study received approval from the Institutional Ethics Committee (approval number 32/KBE/2019).

### 2.2. Population and Sample

Included infants were allocated to the study or control group based on conjugated bilirubin concentration. Patients with a confirmed diagnosis of cholestasis (defined as a conjugated bilirubin concentration > 1.0 mg/dL in accordance with the ESPGHAN 2017 guidelines [3] were eligible for inclusion in the study group. The two additional inclusion criteria were a corrected age of less than 3 months and written informed consent from a parent or legal guardian.

The inclusion criteria for the control group were: corrected age < 3 months; a direct (conjugated) bilirubin concentration < 1.0 mg/dL, regardless of the total bilirubin level; and written informed consent from a parent or legal guardian.

The exclusion criteria applied prior to study initiation (for both the study group and the control group) were: total PN at the time of enrollment (precluding oral administration of vitamins), corrected age above 3 months, multiple organ failure, including hepatic and renal failure, and lack of parental or legal guardian consent for participation in the study.

Term infants were born between 37 and 41 weeks of gestation, whereas preterm infants were born at <37 weeks of gestation.

The etiology of cholestasis was determined based on the final clinical diagnosis established by the treating team and documented in the medical records. For the primary analyses, etiologies were categorized as biliary atresia or other causes of cholestasis. Cholestasis was not separately classified as mild, moderate, or severe, and no validated disease severity scale was used. Instead, biochemical activity of cholestasis was assessed indirectly using direct bilirubin and bile acid concentrations and gamma-glutamyl transferase (GGTP) activity, which were analyzed as continuous variables.

### 2.3. Clinical Data Collection

Clinical data were collected by physicians working at the Department based on interviews with parents or caregivers and a review of medical records. Data were collected at the baseline and follow-up assessments according to a predefined protocol. Treatment adherence was assessed using information from parents or caregivers and available medical records; no objective adherence measure was used.

**Figure 1:**
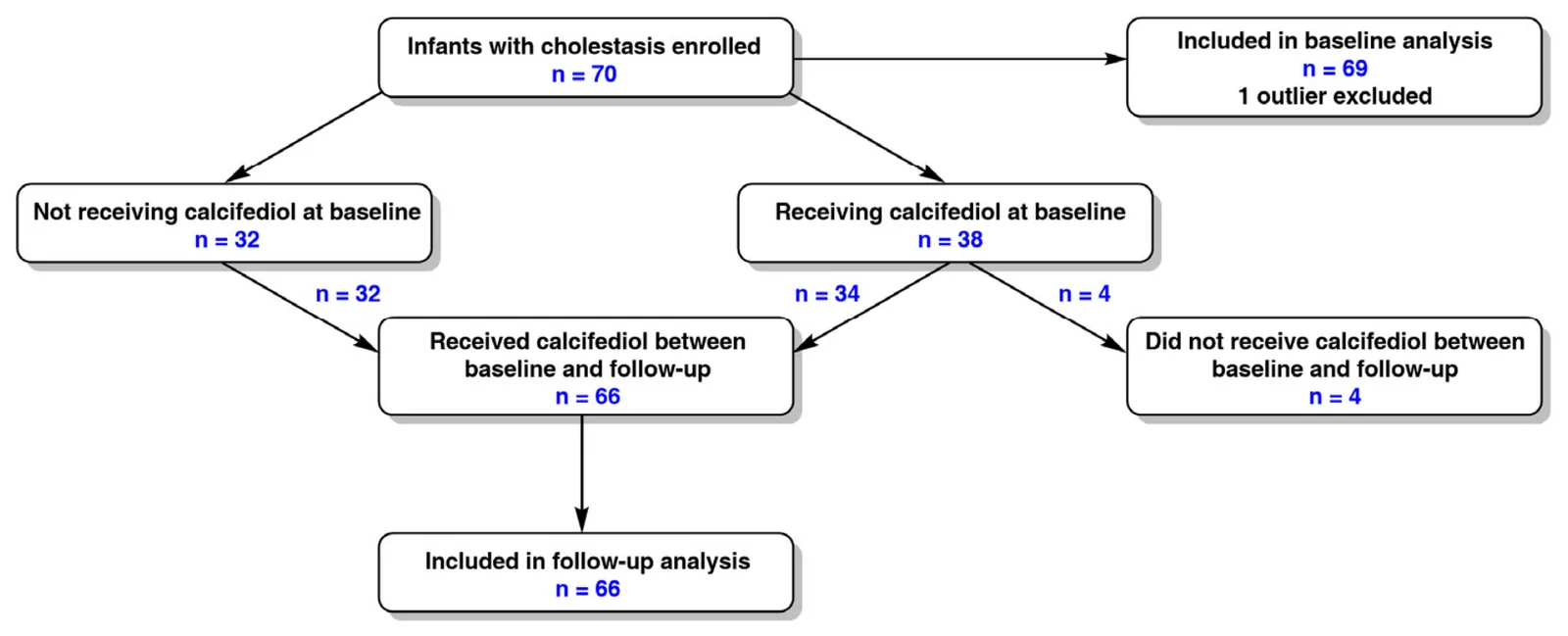
Participant flow and calcifediol exposure in the cholestasis cohort.

The data of family history of diseases, neonatal status at birth (the gestational age at birth (GA), birth weight (BW), mode of delivery, Apgar scores at 1 and 5 min, presence of neonatal jaundice, the onset of cholestasis, duration of total parenteral nutrition, the presence of comorbidities—especially previous infections, necrotizing enterocolitis (NEC), and other surgical interventions were collected. Additionally, current nutritional practices, including dietary intake of fat-soluble vitamins from diet and supplements in the period preceding study enrollment as well as during ongoing supplementation. Baseline vitamin D supplementation reflected treatment prescribed before study enrollment as part of routine clinical care and was not assigned by the investigators. According to the original study protocol, calcifediol was intended to be administered to all infants with cholestasis. At the time of study enrollment, 38 (54%) infants with cholestasis had been receiving calcifediol: 18 (46%) term infants and 20 (64%) preterm infants. The remaining 32 patients had been receiving vitamin D3 supplementation. Following enrollment and review of the baseline serum 25OHD results, calcifediol treatment was continued or initiated in 66 of the 70 infants. In 4 infants, treatment was discontinued because of high baseline serum 25OHD concentrations (Figure 1). Calcifediol was prescribed as Devisol-25 (1 drop = 5 µg of calcifediol; Chema-Elektromet, Rzeszów, Poland), formulated with propylene glycol (E1520) as the excipient, at a dose of 2–5 µg/kg/day in accordance with the recommendations of Baker et al. [20].

Body weight was the only anthropometric parameter consistently available for all participants. Body weight was measured using clinical scales available in our department; body weight was measured without clothing or diapers to the nearest 0.01 kg.

### 2.4. Laboratory Data

Biochemical analyses (total and direct bilirubin; liver enzymes—aspartate aminotransferase (AST), alanine aminotransferase (ALT), gamma-glutamyl transferase (GGT), bile acids, coagulation profile: activated partial thromboplastin time (aPTT), prothrombin time, and international normalized ratio (INR)) were performed in the clinical laboratory of the Children’s Memorial Health Institute, which operates in accordance with institutional standardized quality assurance procedures. Measurement uncertainty (MU) was not estimated separately for this study, as it was designed as a clinical-epidemiological investigation rather than an analytical method validation. Serum 25-hydroxyvitamin D (25OHD) concentration was measured using a fully automated electrochemiluminescence immunoassay (ECLIA) on an IDS analyzer. In accordance with the manufacturer’s instructions, the assay’s analytical measuring range was 4–110 ng/mL, and values below 4 ng/mL were reported as <4 ng/mL. The limit of blank (LoB), limit of detection (LoD), and limit of quantification (LoQ) were 1.31 ng/mL, 1.98 ng/mL, and 3.53 ng/mL, respectively. The total precision of the assay, expressed as the coefficient of variation, ranged from approximately 6.4% to 9.4%. According to the Polish guidelines for prevention and treatment of vitamin D deficiency [18], vitamin D deficiency was defined as serum 25OHD levels <20 ng/mL, an optimal serum level is 30–50 ng/mL, and a high level was defined as a level >50–100 ng/mL.

Clinical assessment of the patients and laboratory investigations were performed twice in the study group—at baseline and after 10–14 days of calcifediol supplementation—and once in the control group at baseline. The follow-up assessment was performed in all infants with cholestasis, including those in whom calcifediol treatment was discontinued or not initiated. No infants were lost to follow-up between the two planned assessments.

### 2.5. Statistical Analysis

Statistical analyses were performed using R version 4.5.2 and RStudio version 2026.01.0 (Posit, Boston, MA, USA) [21,22]. Descriptive statistics are presented as mean ± standard deviation (SD) or as number (percentage), as appropriate. All tests were two-sided, and a *p*-value < 0.05 was considered statistically significant. Between-group comparisons of categorical variables were performed using Pearson’s chi-square test or Fisher’s exact test, as appropriate. Continuous variables were compared using two-sample permutation *t*-tests with 10,000 permutations and unequal variances.

The primary outcome of the present analysis was serum 25OHD concentration. Prespecified primary comparisons included cholestasis versus control infants, preterm versus term infants, and, within the cholestasis group, biliary atresia versus other causes of cholestasis. Longitudinal analyses of follow-up 25OHD concentrations and their associations with baseline 25OHD and calcifediol exposures were considered secondary analyses. These primary and secondary analytical objectives were defined a priori. Analyses of vitamin D concentration categories, additional biochemical parameters, calcium–phosphate metabolism available only in subsets of participants, and interactions between calcifediol exposure and changes in cholestasis biomarkers were considered exploratory. This exploratory classification was defined post hoc, after review of the data, to distinguish these analyses from the prespecified primary and secondary analyses. No formal adjustment for multiple testing was applied; therefore, findings from exploratory analyses were interpreted cautiously and considered hypothesis-generating.

Associations between clinical variables and serum 25-hydroxyvitamin D (25OHD) and other biochemical outcomes were assessed using generalized linear models (GLMs) with a Gamma distribution and a log link, selected because the data were positive and right-skewed. Results are reported as regression coefficients (β) or exponentiated coefficients (expβ) with 95% confidence intervals (95% CI). Model significance was assessed using likelihood ratio tests (type II analysis of deviance). Gestational age at birth was treated as a continuous variable to preserve statistical power and variability. For each outcome, an initial model including study group (cholestasis vs. control), gestational age, and their interaction was fitted. When the interaction term was not significant, a reduced model including only main effects was used. The etiology of cholestasis was not included in these models because they included both infants with cholestasis and control infants, for whom the etiology variable was not applicable. Positive, skewed biochemical outcomes (25OHD, bilirubin fractions, and liver enzymes) were analyzed using Gamma GLMs. Vitamin D intake variables were analyzed using permutation ANCOVA with the Freedman–Lane procedure (9.999 permutations), with corresponding OLS models used for estimation and visualization. One observation identified as an outlier during model diagnostics was excluded from the corresponding regression analysis; this patient was included in all other analyses.

The study did not apply a categorical classification of cholestasis as mild, moderate, or severe, nor did it use a validated clinical or histological scale to assess its severity. The biochemical activity of cholestasis was evaluated using direct bilirubin, bile acid concentrations, and GGTP activity, all analyzed as continuous variables. Direct bilirubin concentration and GGTP activity were included in the multivariable model of follow-up 25OHD concentration, whereas bile acid concentration was included in the comparative analyses. Longitudinal determinants of serum 25OHD at follow-up were analyzed using Gamma GLMs, including baseline 25OHD, dietary vitamin D intake, calcifediol dose, bilirubin and GGT levels, and gestational age. Gestational age was centered at 40 weeks. Competing exposure definitions for calcifediol were compared using the Akaike information criterion (AIC), and cumulative exposure provided the best model fit. Additional models evaluated changes in cholestasis biomarkers over time; interactions were tested but excluded when they did not improve model fit or interpretability.

Model assumptions were assessed using multicollinearity diagnostics (variance inflation factors and condition numbers) and residual analysis (Dunn–Smyth residuals).

## 3. Results

### 3.1. Characteristics of the Study Population

A total of 127 infants were included in the study: 70 infants with cholestasis (39 term infants and 31 preterm infants) and 57 controls (30 term infants and 27 preterm infants). The groups did not differ significantly in terms of mean gestational age (GA), birth weight, postmenstrual age (PMA) at study inclusion, or Apgar score at 1 min of life. However, baseline body weight was significantly lower in infants with cholestasis than in the control group (Table 1).

The mean age at cholestasis onset was 14.53 ± 18.06 days of life, with cholestasis occurring significantly later in term infants than in preterm infants. Biliary atresia was diagnosed in 21 (30%) infants in the study group and was more frequent among term than preterm infants: 15 (38%) vs. 6 (19.3%), respectively; *p* = 0.08. Consistent with the clinical presentation of cholestasis, significant between-group differences were observed in gallbladder appearance on abdominal ultrasound and stool color. An abnormal or non-visualized gallbladder, as well as acholic or variably colored stools, were observed almost exclusively in infants from the study group. Detailed clinical characteristics are presented in Table 1.

### 3.2. Baseline Vitamin D Status and Supplementation

At baseline, dietary vitamin D3 intake was higher in preterm than term infants in both groups, whereas it was comparable between term infants in the study and control groups. The daily dose of supplemental vitamin D3 was significantly higher in preterm infants than in term infants, regardless of whether they belonged to the study or control group. Calcifediol was administered to only 38 (54%) infants with cholestasis, including 18 (46%) term infants and 20 (64%) preterm infants, before recruitment into the study. The mean calcifediol dose was comparable between term and preterm infants. Detailed data are presented in Table 2.

**Figure 2:**
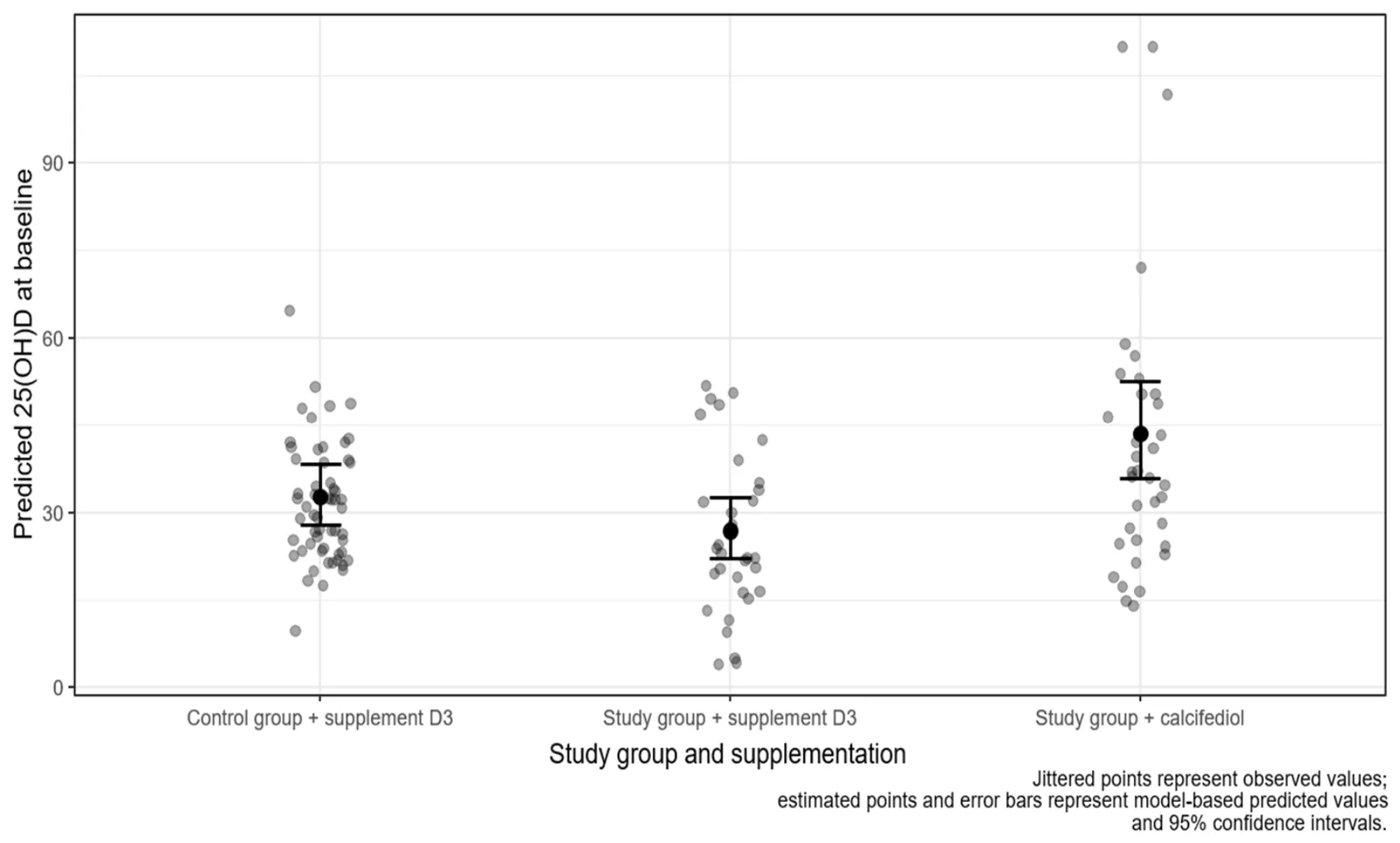
Baseline serum 25OHD concentration by study group and type of vitamin D supplementation.

At baseline, no statistically significant difference in mean serum 25OHD concentrations was found between the study and control groups. In an additional analysis, the study group was stratified by type of vitamin D supplementation, distinguishing infants receiving calcifediol from those receiving standard vitamin D3; both subgroups were then compared with the control group. Infants receiving calcifediol had higher 25OHD concentrations than infants in the study group receiving standard vitamin D3 supplementation and those in the control group. The overall model was statistically significant (*p* < 0.005) (Figure 2).

Among infants with cholestasis, vitamin D deficiency (25OHD < 20 ng/mL) was observed in 16 (22.8%) infants, compared with only 3 (5%) in the control group. Conversely, 25OHD concentrations above the upper limit of optimal range (>50 ng/mL) were observed in 13 (18.6%) infants with cholestasis, 11 (84.6%) of whom had received calcifediol, compared with only 2 (3.5%) infants in the control group (Table 3). Serum 25OHD concentrations exceeding 100 ng/mL, representing a potentially concerning biochemical finding, were observed in three infants with cholestasis. However, because systematically collected concurrent data on calcium–phosphate metabolism, renal function, urinary calcium excretion, and clinical symptoms were not available, these values cannot be interpreted as evidence of vitamin D toxicity.

Baseline 25OHD concentration did not differ significantly between infants with biliary atresia and those with other types of cholestasis, either among preterm or term infants (Table 4).

In the multivariable model, gestational age was not independently associated with baseline 25OHD concentration, whereas the only independent predictors of baseline serum 25OHD concentration were study group and calcifediol (Table 5). The model showed that, compared to infants with cholestasis, controls had a 22.1% higher expected 25OHD concentration, whereas among infants with cholestasis, each 1 µg/kg increase in calcifediol dose was associated with a 15.7% higher expected 25OHD concentration. Finally, a total of 126 infants were included in the multivariable analysis; one infant was excluded because of an extremely high calcifediol dose compared with the remaining patients, which was considered an outlier that could substantially distort model estimates. Detailed data are presented in Table 5.

### 3.3. Vitamin D Status and Supplementation at Follow-Up

**Figure 3:**
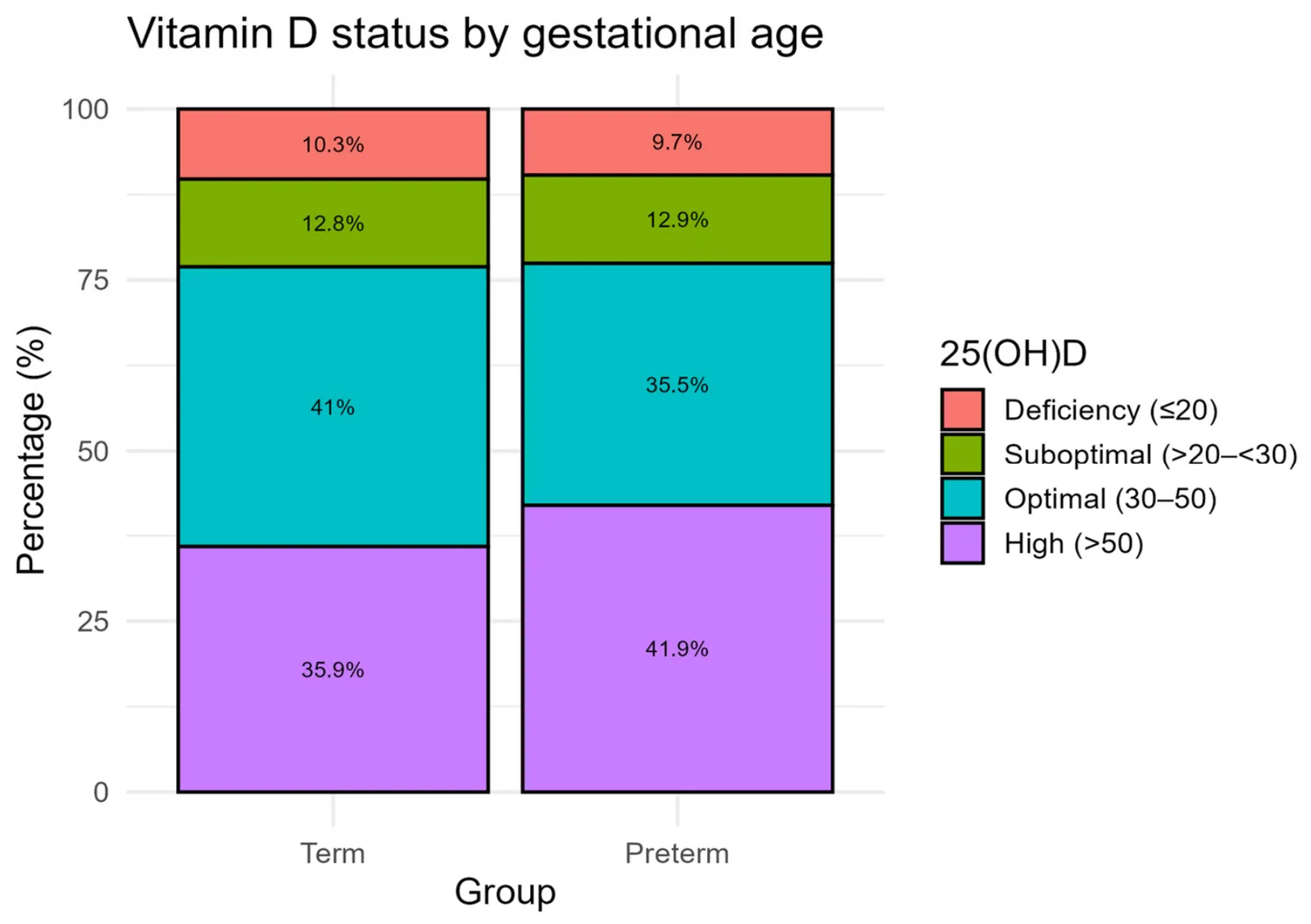
Comparison of vitamin D status between term and preterm infants with cholestasis at follow-up (no significant differences between groups; all *p*-values > 0.05).

At follow-up, dietary vitamin D3 intake remained higher in preterm than in term infants, while calcifediol supplementation did not differ significantly between subgroups (Table 6). Despite this difference, optimal vitamin D status was observed in a similar proportion of term and preterm infants (41% vs. 35.4%, respectively; *p* = 0.636). Detailed data are presented in Figure 3.

Among infants with biliary atresia, preterm infants had lower 25OHD concentrations than term infants, although the difference was not statistically significant (Table 4).

Additional data on calcium–phosphate metabolism were available for a subset of infants with cholestasis. At baseline, ALP activity measurements were available for 44 infants, including 21 preterm and 23 term infants, while serum calcium and phosphate concentrations were available for 60 and 61 infants, respectively. Preterm infants had higher total ALP activity and lower serum calcium than term infants, whereas phosphate concentrations did not differ significantly between the groups. At follow-up, ALP data were available for 32 infants, including 17 preterm and 15 term infants. Total ALP activity did not differ significantly between preterm and term infants. Serum calcium and phosphate concentrations were available for 49 and 50 patients, respectively. Preterm infants had lower serum phosphate concentrations, whereas the difference in calcium concentrations did not reach statistical significance.

Additionally, at baseline, two patients had hypercalcemia (Ca > 2.75 mmol/L), and one patient had hyperphosphatemia (*p* > 2.7 mmol/L). At follow-up, two patients had hypercalcemia, whereas no cases of hyperphosphatemia were observed. Because these data were available only for a subset of participants, these analyses were considered exploratory. Detailed results are presented in Table 7.

To identify predictors of follow-up 25OHD concentration in infants with cholestasis, a generalized linear model with a Gamma distribution and a log-link function was applied. The analysis included only infants receiving calcifediol (*n* = 66); all four infants excluded from the calcifediol exposure-specific regression analysis completed the follow-up assessment and were included in the other descriptive analyses.

In the multivariable model, baseline 25OHD concentration and post-enrollment cumulative calcifediol exposure—calculated as the daily dose multiplied by the number of days between assessments—were the only variables independently associated with follow-up 25OHD concentration.

**Figure 4:**
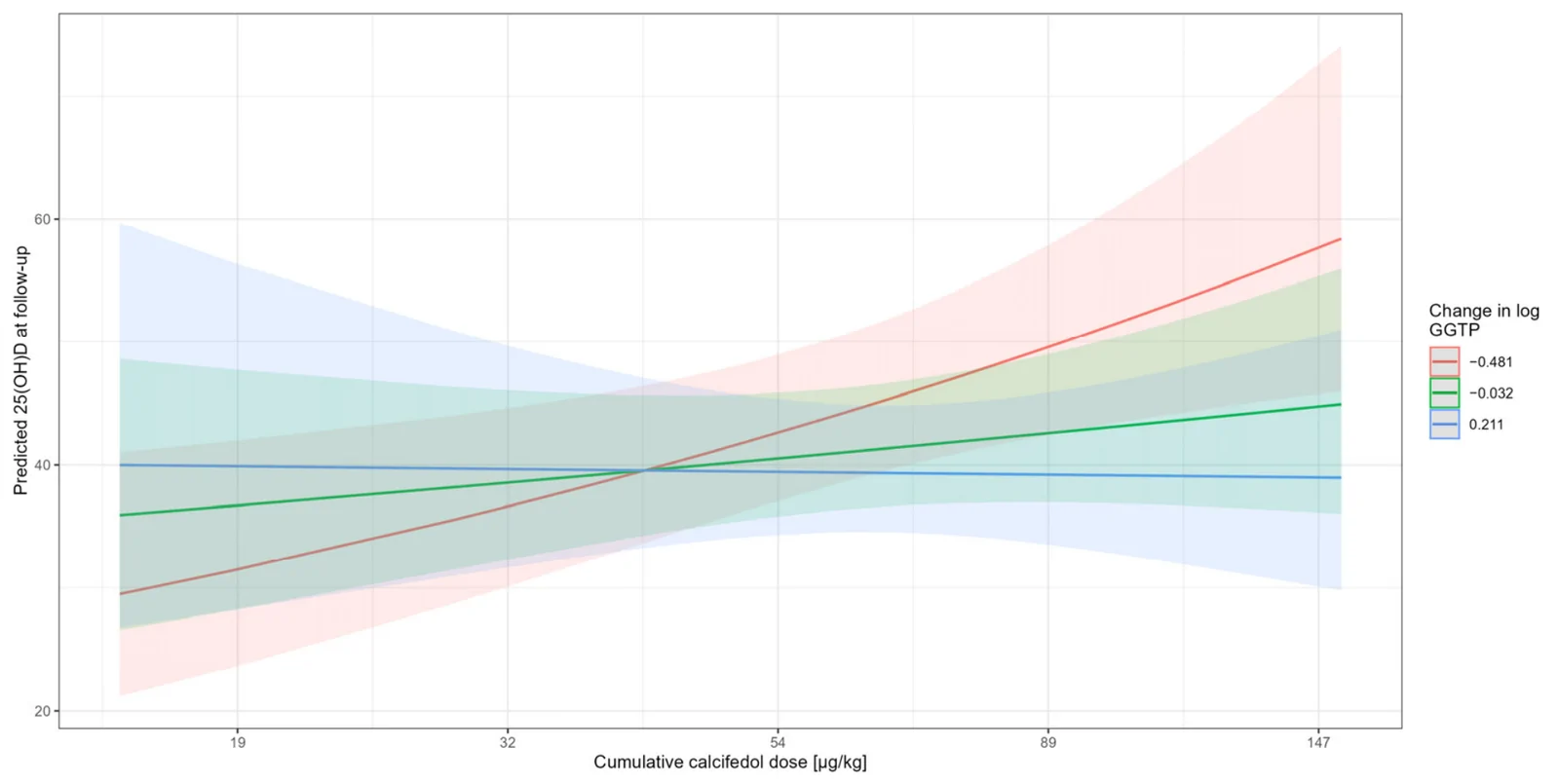
Association between cumulative calcifediol exposure and predicted follow-up 25OHD concentration according to changes in GGTP activity in infants with cholestasis.

After logarithmic transformation, doubling cumulative calcifediol exposure was associated with an expected 25OHD concentration approximately 19.87% higher at follow-up (Table 8). In this cohort, no statistically significant independent association was detected between gestational age and follow-up 25OHD concentration after adjustment for the other variables included in the model. In an additional exploratory analysis, we assessed whether the association between cumulative calcifediol exposure and follow-up 25OHD concentration differed according to changes in biochemical markers of cholestatic activity. No evidence of an interaction was observed for changes in direct bilirubin concentration (*p* = 0.913). For changes in GGTP activity, the interaction trended toward statistical significance (*p* = 0.054). The observed pattern suggested a possible weaker positive association between cumulative calcifediol exposure and follow-up 25OHD concentration in infants with increasing GGTP activity. This finding should be interpreted as exploratory. The pattern of model-predicted values is presented in Figure 4; given the non-significant interaction, it should be interpreted as exploratory and hypothesis-generating.

## 4. Discussion

In the present study, we evaluated vitamin D intake and serum 25OHD concentration in infants with cholestasis, taking into account gestational maturity, type of supplementation, etiology of cholestasis, and selected biochemical parameters. The analysis identified three main clinically relevant observations. First, vitamin D status in this population was characterized by substantial heterogeneity, with both low and elevated 25OHD concentrations observed. This finding indicates considerable variability in the response to supplementation and underscores the need for individualized treatment and regular monitoring. Second, in contrast to most previous studies, which focused primarily on infants with biliary atresia (BA) or on children with chronic liver disease [2,10,13,14,15,16,17,18], our study also considered the impact of gestational maturity. Despite the biologically increased risk of vitamin D deficiency in preterm infants, there was no independent association between gestational maturity and 25OHD concentration in the adjusted analyses. Third, we identified factors independently associated with 25OHD concentration in infants with cholestasis. The most important predictors were the presence of cholestasis, baseline 25OHD concentration, and exposure to calcifediol treatment. In addition, the exploratory analysis of changes in GGTP activity suggested a possible variation in the association between cumulative calcifediol exposure and follow-up 25OHD concentration. However, because the interaction did not reach statistical significance (*p* = 0.054), this observation should be interpreted cautiously. The principal outcome of our study was serum 25OHD concentration. However, serum 25OHD concentration alone does not provide a comprehensive assessment of bone mineralization and cannot be used to diagnose or exclude metabolic bone disease. The study was not designed to evaluate calcium–phosphate metabolism or metabolic bone disease in preterm infants. Consequently, measurements of PTH, calcium, phosphate, total ALP activity, and ALP isoenzymes were not prespecified in the protocol as mandatory assessments. Routinely obtained results for total ALP activity and serum calcium and phosphate concentrations were available only for a subset of participants and were therefore included as exploratory data. These issues should be evaluated in a separately designed prospective study incorporating mandatory serial measurements of PTH, calcium, phosphate, total and bone-specific ALP, and other markers of bone turnover. In our cohort of infants with cholestasis, only 38.5% of infants achieved an optimal 25OHD concentration (30–50 ng/mL) [18] although the supplemental dose of calcifediol was within the recommended range [20]. At the same time, another 38.5% of patients had 25OHD concentration > 50 ng/mL, including three children with values exceeding 100 ng/mL. Vitamin D deficiency, defined as a 25OHD concentration < 20 ng/mL, was observed in 16 of 70 infants, corresponding to 22.8% of the study group. These findings indicate a highly heterogeneous response to treatment: in some patients, deficiency persisted despite supplementation, whereas in others, high 25OHD concentration was achieved. The bidirectional variability described above is consistent with available literature data [1,17,19,21]. From a clinical perspective, these findings indicate the need not only to select an appropriate supplementation strategy, but also to regularly monitor 25OHD concentration and dynamically adjust the dose.

Vitamin D deficiency is very common among infants with cholestasis, particularly in those with BA, with a reported prevalence of approximately 61–88% [22,23,24,25,26], mainly due to insufficient monitoring and low rates of vitamin D supplementation.

In our analysis, the prevalence of vitamin D deficiency was markedly lower than in the above-mentioned studies. Differences in the prevalence of vitamin D deficiency between our study and previous reports may be explained by the inclusion of infants with biliary atresia as well as those with other types of cholestasis in our cohort, making the study population more heterogeneous. In addition, 54% of infants received calcifediol, which, as demonstrated in our regression analysis, was significantly and positively associated with serum 25OHD concentration. The higher 25OHD concentrations observed in infants receiving calcifediol should be interpreted with caution. The comparison with infants receiving vitamin D3 was observational and non-randomized, and the control group differed fundamentally from the cholestasis group with respect to the underlying disease and clinical condition. Rather, these findings indicate an association between calcifediol exposure and higher measured 25OHD concentrations in the studied cohort. This interpretation is further supported by the multivariable longitudinal analysis, in which cumulative calcifediol exposure was independently associated with higher follow-up 25OHD concentrations. However, the influence of other confounding factors related to disease characteristics, treatment indications, nutritional status, or other unmeasured factors cannot be excluded. A direct comparison of the efficacy of calcifediol and vitamin D3 in infants with cholestasis would require prospective randomized studies. The relevance of this finding may be explained by calcifediol’s properties. Importantly, calcifediol does not require hepatic 25-hydroxylation, as it is already the metabolite formed after this step of vitamin D metabolism. As a result, it may increase serum 25OHD concentration more rapidly and more predictably, which is particularly relevant in children with liver disease and impaired bile flow [27,28,29]. In the present study, intestinal absorption, bioavailability, and pharmacokinetic parameters were not assessed; therefore, any potential advantage of calcifediol over vitamin D3 in these parameters remains a biologically plausible hypothesis that cannot be confirmed on the basis of our findings.

The type of vitamin D preparation used (D2, D3, or calcifediol) may be relevant to treatment response. In our study, infants receiving calcifediol had higher observed 25OHD concentrations than those receiving vitamin D3; however, given the observational and non-randomized study design, these findings should not be interpreted as evidence of greater efficacy of calcifediol.

Jensen et al. [30] reported that oral ergocalciferol failed to raise 25OHD concentrations above 20 ng/mL in any infant with cholestasis. Similarly, Mariano da Rocha et al. [31] showed that high-dose cholecalciferol (6000 IU/day) increased 25OHD concentration in only 29% of patients. Ng et al. [32] also concluded that standard supplementation is insufficient in this population. Shneider et al. [16] showed that even multivitamin formulations designed to enhance absorption did not normalize vitamin D status, with deficiency persisting in 37% of patients with BA after hepatoportoenterostomy. This conclusion is further supported by Chongthavornvansa et al. [2], who demonstrated that even very high doses of vitamin D2 resulted in only a modest increase in 25OHD concentration, particularly in infants with biliary atresia. Although both ergocalciferol (vitamin D2) and cholecalciferol (vitamin D3) are converted to circulating 25OHD, they differ in pharmacokinetics and biological potency. Pediatric data remain limited, but available studies suggest that vitamin D3 may produce greater increases in 25OHD concentrations than equivalent doses of vitamin D2, although in some settings both forms may exert comparable effects [32,33,34,35]. Gordon et al. [32] found no short-term differences between high-dose D2 and D3 in healthy infants, whereas a randomized trial in children with inflammatory bowel disease demonstrated superior efficacy of daily vitamin D_3_ compared with vitamin D2 [34].

Overall, the available evidence confirms that pediatric cholestasis is associated with a high risk of disturbances in fat-soluble vitamin status. At the same time, however, there is no single universal supplementation regimen suitable for all patients. Management should therefore be individualized, taking into account both the risk of deficiency and the possibility of an excessive 25OHD concentration. This view has been emphasized by Degrassi et al. [1], Kamath et al. [11], Socha and Pawłowska [12], Mouzaki et al. [19], and Veraldi et al. [23].

To the best of our knowledge, previous studies have not directly analyzed the combined impact of prematurity and cholestasis on vitamin D status. Ukarapong et al. [13] showed that baseline 25OHD concentration was similar in preterm infants with and without cholestasis, whereas at the second measurement point, they were lower in the cholestasis group, despite a comparable prevalence of deficiency. Dong et al. [24] reported more severe 25OHD deficiency in younger patients with biliary atresia before surgical treatment, and Mariano da Rocha et al. [31] showed that children with vitamin D deficiency were younger and had more advanced liver disease. In turn, Saron et al. [36] described an association between anthropometric indices and vitamin concentration.

In the context of our findings, these observations are clinically relevant, as they suggest that abnormal vitamin D status in infants with cholestasis may be associated with poorer nutritional status and limited metabolic reserves. Prematurity, through gastrointestinal immaturity, lower body stores, and greater susceptibility to nutritional disturbances, could therefore exacerbate the adverse effect of cholestasis on vitamin D status.

In our analysis, however, gestational maturity was not an independent predictor of 25OHD concentration, despite our initial assumption that preterm infants with cholestasis would be at a particularly high risk of vitamin D deficiency.

However, this finding should be interpreted cautiously. Only 31 preterm infants with cholestasis were included in the study, which limited the statistical power to detect an independent association with gestational maturity. In addition, the absence of such an association may be explained by the fact that preterm infants received higher vitamin D3 intake from both diet and supplementation and were more frequently treated with calcifediol, which, as indicated above, showed a strong association with serum 25OHD concentration. Although the mean calcifediol dose was comparable between preterm and term infants, the higher proportion of preterm infants receiving this preparation may have partially offset their greater biological susceptibility to deficiency. After adjustment for more direct determinants of vitamin D status, such as baseline 25OHD concentration and calcifediol exposure, gestational maturity itself may no longer have shown an independent effect. This finding corresponds with the lack of statistically significant differences in final 25OHD concentration between preterm and term infants.

It should also be emphasized that measurement of 25OHD concentration alone does not fully reflect the risk of metabolic bone disease, which is an important comorbidity in preterm infants with cholestasis. Vitamin D deficiency may lead to impaired bone mineralization [37,38], but skeletal complications in chronic liver disease are multifactorial and depend not only on vitamin D status, but also on calcium and phosphate intake, liver disease activity, nutritional status, muscle mass, physical activity, and treatment used. Therefore, future studies should include parallel assessment of PTH, calcium, phosphate, alkaline phosphatase activity, bone turnover markers, and—in selected patient groups—bone mineralization status [13,39].

In the present study, cumulative calcifediol exposure was independently associated with higher follow-up 25OHD concentration. Among the evaluated measures of calcifediol exposure, cumulative exposure provided the best model fit, suggesting that the overall exposure during the supplementation period may be relevant to the achieved 25OHD concentration. These findings support regular monitoring of 25OHD and individualized adjustment of supplementation. In the multivariable analysis, neither direct bilirubin concentration nor GGTP activity was an independent predictor of 25OHD concentration. An exploratory analysis of changes in GGTP activity showed a pattern suggesting that the positive association between cumulative calcifediol exposure and follow-up 25OHD concentration may be less pronounced in infants with increasing GGTP activity. However, as the interaction did not reach statistical significance (*p* = 0.054), this observation should be considered preliminary.

Published data on the association between 25OHD concentration and biochemical markers of cholestasis remain inconsistent. Mariano da Rocha et al. [31] demonstrated an association between vitamin D deficiency and higher total bilirubin concentration, GGTP activity, and alkaline phosphatase (ALP) activity, whereas Zhuang et al. [25] observed an association only with ALP and markers of fibrosis. Ng et al. [26] reported correlations between 25OHD concentration and bilirubin, ALP, and phosphate levels after portoenterostomy, particularly in children with persistent jaundice. Venkat et al. [6] identified total bilirubin as a useful predictor of fat-soluble vitamin deficiency in infants with biliary atresia, while Fangjuan et al. [12] suggested that low 25OHD concentration may have diagnostic value in identifying biliary atresia.

A key strength of this study is the inclusion of preterm infants, a group rarely examined in the context of cholestasis despite their high susceptibility to vitamin D deficiency due to limited fetal stores, organ immaturity, and greater nutritional vulnerability. Most previous research [2,16,23,24,25,26,27,28] has focused on term infants with biliary atresia or chronic liver disease, without evaluating the specific contribution of prematurity. Our findings, therefore, expand current evidence by analyzing vitamin D status separately in preterm and term infants. Although preterm infants received a higher vitamin D intake, this did not translate into higher 25OHD concentrations, and no independent association between gestational age and 25OHD concentration was detected in the adjusted analyses. Clarification of the independent effect of gestational maturity requires larger prospective studies with more comparable supplementation regimens.

In summary, the present study demonstrates the marked heterogeneity in responses to vitamin D supplementation among infants with cholestasis. Both persistent 25OHD deficiency and high 25OHD concentrations were observed, particularly in patients receiving calcifediol. Although preterm infants are biologically more susceptible to vitamin D deficiency, no independent association between gestational maturity and 25OHD concentration was detected in the present cohort. However, this finding should be interpreted with caution because of the limited number of preterm infants and differences in vitamin D intake and in the frequency of calcifediol use between preterm and term infants. Cumulative calcifediol exposure was independently associated with higher follow-up 25OHD concentration. Together with the substantial interindividual variability observed, these findings support individualized supplementation based on regular monitoring of 25OHD concentration and careful adjustment of treatment.

This study also has several limitations. First, the group of infants with cholestasis was heterogeneous, including infants with biliary atresia and other types of cholestasis, such as transient cholestasis, which reflects clinical practice but may limit etiologically specific conclusions. The relatively small number of preterm infants with cholestasis limited the statistical power to detect an independent association between gestational maturity and 25OHD concentration, particularly in the context of differences in vitamin D intake and calcifediol exposure between preterm and term infants. In addition, markers of bone turnover, body composition, and liver fibrosis were not assessed, limiting evaluation of skeletal consequences and the ability to conclude the therapy’s clinical safety.

Finally, genetic determinants of vitamin D metabolism were not analyzed, which may at least partly explain observed variability in 25OHD concentration.

The observational design and the absence of randomized allocation to supplementation type should also be considered when interpreting differences between calcifediol and vitamin D3 supplementation. An additional limitation concerns cumulative calcifediol exposure, which included only the post-enrollment period. In infants already receiving calcifediol at enrollment, pre-enrollment treatment duration was not systematically recorded and therefore could not be included, potentially underestimating true total exposure. Although baseline 25OHD concentration may partially reflect prior treatment, residual confounding cannot be excluded. The resulting bias could have either overestimated or attenuated the observed association with follow-up 25OHD concentration, depending on prior treatment duration and subsequent dose changes. Furthermore, cholestasis severity was not assessed using a validated clinical or histological scale; therefore, direct bilirubin and GGTP were interpreted as biochemical markers of cholestatic activity. The interaction involving changes in GGTP activity did not reach statistical significance and should be considered exploratory. In addition, multiple statistical comparisons were performed without formal adjustment for multiplicity, which increases the risk of type I error; therefore, in particular, the exploratory subgroup and interaction analyses should be interpreted as hypothesis-generating and require confirmation in larger independent cohorts. Treatment adherence was not formally assessed using a standardized or objective method, and this has been acknowledged as a limitation of the study.

## 5. Conclusions

Vitamin D status in infants with cholestasis is highly variable in both term and preterm infants, and gestational maturity was not independently associated with 25OHD concentration. Cumulative calcifediol exposure was independently associated with higher follow-up 25OHD concentrations; however, the observational, non-randomized design does not allow direct comparison of the efficacy of calcifediol versus vitamin D3 supplementation. An exploratory analysis suggested a possible trend toward a weaker association between cumulative calcifediol exposure and follow-up 25OHD concentration in infants with increasing GGTP activity; however, the interaction did not reach statistical significance (*p* = 0.054). The substantial interindividual variability in 25OHD concentrations supports regular monitoring and individualized adjustment of supplementation. However, the limited sample size and the absence of a comprehensive assessment of disease severity do not allow for clinically relevant associations between cholestasis severity and vitamin D status.

## Figures and Tables

**Table 1:** Baseline characteristics and biochemical markers of the study and control groups and a comparison between preterm infants and term infants. Data are presented as the mean +/− SD or number (n%). The statistically significant *p* value is <0.05.

Variable	Total (*n* = 127)	Study Group (*n* = 70) Term Infants (*n* = 39) Preterm Infants (*n* = 31)	*p*-Value (Term vs. Preterm)	Control Group (57) Term Infants (*n* = 30) Preterm Infants (*n* = 27)	*p*-Value (Term vs. Preterm)	*p*-Value (Study vs. Control)
Gestational age (wks)	35.11 ± 4.93	35.17 ± 5.09 Term: 38.82 ± 1.27 Preterm: 30.58 ± 4.30	<0.001	35.04 ± 4.72 Term: 38.73 ± 1.41 Preterm: 30.93 ± 3.67	<0.001	0.882
Birth weight (g)	2496 ± 1012	2413 ± 954.0 Term: 3030 ± 554.6 Preterm: 1637 ± 765.9	<0.001	2598 ± 1081 Term: 3420 ± 572.7 Preterm: 1685 ± 708.0	<0.001	0.312
Apgar at 1′	8.35 ± 2.47	8.01 ± 2.79 Term: 9.69 ± 0.77 Preterm: 5.90 ± 2.99	<0.001	8.75 ± 1.95 Term: 9.90 ± 0.31 Preterm: 7.48 ± 2.21	<0.001	0.082
PMA (wks)	41.17 ± 3.34	40.99 ± 3.03		41.39 ± 3.69		0.512
Body weight (g) at baseline	3442 ± 923.9	3160 ± 709.0 Term: 3435 ± 698.3 Preterm: 2814 ± 563.2	<0.001	3741 ± 1063 Term: 4471 ± 753.3 Preterm: 2931 ± 710.5	<0.001	<0.001
Type of delivery, *n* (%): -cesarean section -natural birth	75 (59%) 52 (41%)	44 (63%) 26 (37%)		31 (54%) 26 (46%)		0.433
Presence of jaundice, *n* (%): -No -Yes	30 (24%) 97 (76%)	11 (16%) 59 (84%)		19 (33%) 38 (67%)		0.034
Phototherapy: -No -Yes	87 (69%) 40 (31%)	43 (61%) 27 (39%)		44 (77%) 13 (23%)		0.087
Gallbladder on US -normal -small (contracted) -absence or abnormal	71 (66%) 7 (6.5%) 29 (27%)	35 (50%) 6 (8.6%) 29 (41%)		36 (97%) 1 (2.7%) 0 (0%)		<0.001
Stools -fluctuating -pale -normal	10 (7.9%) 23 (18%) 94 (74%)	10 (14%) 23 (33%) 37 (53%)		0 (0%) 0 (0%) 57 (100%)		<0.001
Onset of cholestasis (day of life)		14.53 ± 18.06 Term (*n* = 39): 21.71 ± 21.39 Preterm (*n* = 31): 8.82 ± 12.48	0.005	not applicable		
TPN: -no -yes	84 (67%) 42 (33%)	44 (64%) 25 (36%) Preterm 22 (31%)		40 (70%) 17 (30%)		0.569
Type of diet: -breast milk -formula -mix	65 (51%) 44 (35%) 18 (14%)	32 (46%) 22 (31%) 16 (23%)		33 (58%) 22 (39%) 2 (3.5%)		0.008
Type of cholestasis: -biliary atresia -other cholestasis	21 (17%) 106 (83%)	21 (30%) Term *n* = 15 (38%) Preterm *n* = 6 (19.4%) 49 (70%) Term *n* = 24 (61.5%) Preterm *n* = 25 (80.6%)	0.08	not applicable		
Total bilirubin (mg/dL)	6.32 ± 4.99	7.87 ± 5.09		4.42 ± 4.17		<0.001
Direct bilirubin (mg/dL)	3.18 ± 3.94	5.41 ± 4.12		0.43 ± 0.18		<0.001
GGTP (U/L)	286.45 ± 335.73	437.23 ± 388.88		101.28 ± 62.97		<0.001
ASP/AT (U/L)	97.89 ± 142.08	144.61 ± 176.87		40.51 ± 28.16		<0.001
AL/AT (U/L)	59.16 ± 88.22	87.24 ± 110.60		24.67 ± 15.56		<0.001
Bile acids (umol/L)		121.30 ± 68.46 Term: 120.34 ± 73.61 Preterm: 122.49 ± 62.74	0.89	not measured		

Abbreviations: wks—weeks, PMA—postmenstrual age, US—ultrasonography, TPN—total parenteral nutrition, GGTP—gamma-glutamyl transpeptidase, ASP/AT—aspartate aminotransferase, AL/AT—alanine aminotransferase.

**Table 2:** Comparison of baseline vitamin D3 and calcifediol intake and serum 25OHD concentrations between the study and control groups and between term and preterm infants.

Variable	Study Group	*p*-Value (Term vs. Preterm)	Control Group	*p*-Value (Term vs. Preterm)	*p*-Value (Study vs. Control)
Vitamin D3—diet (IU/d)	Total (*n* = 70): 210.9 ± 190.9 Term (*n* = 39): 113.29 ± 135 Preterm (*n* = 31): 333 ± 180	0.001	Total (*n* = 57): 266.8 ± 253 Term (*n* = 30): 84.29 ± 157 Preterm (*n* = 27): 469.6 ± 171	0.001	0.171
Vitamin D3 supplementation, *n* (%)	Total *n* = 32 (45.7%) Term *n* = 21 (53.8%) Preterm *n* = 11 (35.5%)	0.126	Total *n* = 57 (100%)		<0.001
Vitamin D3 supplementation (IU/day)	Total (*n* = 32): 615.63 ± 231 Term (*n* = 21): 480.9 ± 87.2 Preterm (*n* = 11): 872.7 ± 200	<0.001	Total (*n* = 57): 592.9 ± 301 Term (*n* = 30): 400 ± 0 Preterm (*n* = 27): 807.41 ± 323	0.001	0.693
Calcifediol, *n* (%)	Total *n* = 38 (54.3%) Term *n* = 18 (46.2%) Preterm *n* = 20 (64.5%)	0.126	not applicable		
Calcifediol (µg/day)	Total (*n* = 38): 11.32 ± 8.03 Term (*n* = 18): 11.39 ± 4.79 Preterm (*n* = 20): 11.25 ± 10.24	0.957	not applicable		
Calcifediol (µg/kg/day)	Total (*n* = 38): 3.70 ± 3.70 Term (*n* = 18): 3.11 ± 0.96 Preterm (*n* = 20): 4.23 ± 5.02	0.357	not applicable		
25OHD (ng/mL)	Total (*n* = 70): 34.81 ± 21.33 Term (*n* = 39): 33.44 ± 22.89 Preterm (*n* = 31): 36.53 ± 19.42	0.543	Total (*n* = 57): 31.55 ± 10.14 Term (*n* = 30): 32.03 ± 9.09 Preterm (*n* = 27): 31.03 ± 11.35	0.717	0.261

**Table 3:** Baseline vitamin D status in the study population and comparison between study and control groups, and between term and preterm infants.

25OHD Concertation (ng/mL)	Total *n* = 127	Study Group *n* = 70	Study Group *n* = 70		Control Group *n* = 57	Control Group	
			Term *n* = 39	Preterm *n* = 31		Term *n* = 30	Preterm *n* = 27
Deficiency (≤20 ng/mL)	19 (15%)	16 (22.8%)	11 (28.4%)	5 (16.1%)	3 (5%)	1(3.3%)	2 (7.4%)
Suboptimal (20–30 ng/mL)	41 (32%)	16 (22.8%)	10 (25.6%)	6 (19.3%)	25 (44%)	12 (40%)	13 (48.1%)
Optimal (30–50 ng/mL)	52 (41%)	25 (35%)	12 (30.7%)	13 (41.9%)	27 (47.5%)	16 (53.4%)	11 (40.7%)
High (≥50–75 ng/mL)	12 (9.4%)	10 (14.3%)	4 (10.2%)	6 (19.3%)	2 (3.5%)	1 (3.3%)	1 (3.8%)
Very high ≥ 75 ng/mL	3 (2.3%)	3 (4.3%)	2 (5.1%)	1 (3.2%)	0 (0%)	0 (0%)	0 (0%)

Data are presented as n (%). Because of the small number of infants in several categories after stratification by study group and gestational maturity, no inferential statistical comparisons were performed for individual 25OHD categories.

**Table 4:** Vitamin D status and biochemical parameters according to the type of cholestasis at baseline and follow-up in term and preterm infants.

Variable	Preterm Infants *n* = 31		*p*-Value	Term Infants *n* = 39		*p*-Value	Total *n* = 70
	Biliary Atresia *n* = 6	Other Cholestatic Etiologies *n* = 25		Biliary Atresia *n* = 15	Other Cholestatic Etiologies *n* = 24		
Baseline GGTP, U/L	630.50 ± 290.18	284.08 ± 214.32	0.031	582.00 ± 452.10	457.96 ± 462.5	0.415	437.23 ± 388.88
Follow-up GGTP, U/L	1003 ± 865.87	295.76 ± 237.90	0.102	670.27 ± 557.96	373.17 ± 403.85	0.086	463.19 ± 489.08
Baseline Direct bilirubin, mg/dL	4.92 ± 1.40	6.49 ± 5.23	0.198	4.16 ± 1.47	5.20 ± 4.29	0.284	5.41 ± 4.12
Follow-up Direct bilirubin (mg/dL)	5.30 ± 2.72	3.71 ± 2.45	0.232	4.78 ± 1.17	3.60 ± 2.79	0.078	4.04 ± 2.42
Baseline 25OHD (ng/mL)	28.23 ± 14.33	38.52 ± 20.18	0.177	33.40 ± 18.83	33.46 ± 25.49	0.993	34.81 ± 21.33
Follow-up 25OHD (ng/mL)	42.40 ± 11.76	46.53 ± 20.20	0.522	37.37 ± 13.90	48.39 ± 22.76	0.068	44.85 ± 19.55
Baseline bile acid (umol/L)	139.47 ± 100.00	118.07 ± 50.94	0.634	132.95 ± 95.56	112.31 ± 56.61	0.474	121.30 ± 68.46
Follow-up bile acid (umol/L)	191.37 ± 152.11	136.77 ± 89.38	0.601	154.90 ± 49.69	119.08 ± 72.96	0.115	137.61 ± 80.07

**Table 5:** Multiplicative effects estimated from the regression model for baseline 25OHD concentration.

Predictor	exp(β) ^f^	95% CI	*p*-Value ^e^
Intercept	25.146	[20.85; 30.427]	<0.001
Control group vs. study group (reference: study group) ^a,b^	1.221	[1.015; 1.465]	0.036
Gestational age (weeks) ^c^	1.008	[0.987; 1.028]	0.483
Calcifediol (µg/kg/day) in study group ^d^	1.176	[1.095; 1.263]	<0.001
Vitamin D3—diet (IU/kg)	1.000	[0.999; 1.002]	0.737
D3—supplements (IU/kg)	1.000	[0.999; 1.001]	0.568

^a^ *n* = 126 infants were included in the analysis. ^b^ The reference category for the variable defining study group was the group of infants with cholestasis (study group). Therefore, the coefficient labeled control group versus study group represents the ratio of the expected 25(OH)D concentration in the control group to the expected concentration in infants with cholestasis, assuming that all other variables included in the model are held constant. ^c^ Gestational age was centered at 40 weeks of gestation; the coefficient describes the change in expected 25OHD concentration associated with a 1-week increase in gestational age. ^d^ Calcifediol dose was included in its original scale, expressed as µg/kg. Calcifediol was administered only in the study group; therefore, its coefficient in the whole-sample model is identified by dose variability among infants with cholestasis and does not represent a between-group comparison of the drug effect. ^e^ Overall model significance: F (5,120) = 5.72, *p* < 0.001. ^f^ Deviance-based pseudo-R^2^ = 0.179; AIC = 1026.6; dispersion parameter = 0.196. exp(β) is interpreted as the ratio of expected 25OHD values, with all other predictors held constant.

**Table 6:** Vitamin D intake and serum 250HD concentration in infants with cholestasis at follow-up.

Variable	Study Group *n* = 70	Term *n* = 39	Preterm *n* = 31	*p*-Value
Vitamin D3—diet (IU/day)	260.96 ± 216	170.53 ± 173	374.72 ± 214	< 0.001
Calcifediol (µg/day)	11.86 ± 5.12	12.31 ± 5.11	11.29 ± 5.16	0.412
Calcifediol (µg/kg/day)	3.87 ± 1.64	3.72 ± 1.62	4.05 ± 1.69	0.412
25OHD ng/mL	44.85 ± 19.55	44.15 ± 20.36	45.73 ± 18.77	0.737

**Table 7:** Calcium–phosphate metabolism parameters in the study population and comparison between term and preterm infants.

Variable	Study Group Baseline	*p*-Value (Term vs. Preterm)	Study Group Follow-Up	*p*-Value (Term vs. Preterm)
ALP (U/L)	Total (*n* = 44): 580.61 ± 29.80 Term (*n* = 23): 486.65 ± 211.32 Preterm (*n* = 21): 683.52 ± 207.70	0.0033	Total (*n* = 32): 582.00 ± 193.50 Term (*n* = 15): 533.33 ± 197.63 Preterm (*n* = 17): 624.94 ± 184.91	0.193
Ca (mmol/L)	Total (*n* = 60): 2.51 ± 0.14 Term (*n* = 30): 2.55 ± 0.13 Preterm (*n* = 30): 2.47 ± 0.13	0.020	Total (*n* = 49): 2.51 ± 0.22 Term (*n* = 25): 2.56 ± 0.20 Preterm (*n* = 24): 2.45 ± 0.22	0.074
*p* (mmol/L)	Total (*n* = 61): 1.99 ± 0.27 Term (*n* = 31): 2.01 ± 0.31 Preterm (*n* = 30): 1.97 ± 0.22	0.563	Total (*n* = 50): 2.09 ± 0.20 Term (*n* = 26): 2.18 ± 0.19 Preterm (*n* = 24): 2.01 ± 0.19	0.003

**Table 8:** The multivariable Gamma regression model for follow-up 25OHD concentrations in 66 infants with cholestasis treated with calcifediol between baseline and follow-up.

Predictor	exp(β)	95% CI	% Change	*p*-Value
Intercept	8.355	(3.141; 22.788)	735.5% (214.1%; 2178.8%)	<0.001
log(25OHD baseline)	1.344	(1.14; 1.578)	34.4% (14.2%; 57.8%)	<0.001
log(1 + cumulative calcifediol dose [µg/kg]) ^a,b^	1.299	(1.058; 1.588)	29.9% (5.8%; 58.8%)	0.016
Vitamin D3—diet (IU/kg)	1.000	(0.999; 1.002)	0% (−0.1%; 0.2%)	0.801
log(direct bilirubin + 0.1)	0.969	(0.829; 1.132)	−3.1% (−17.1%; 13.2%)	0.687
log(GGTP 1 + 1)	0.942	(0.844; 1.052)	−5.8% (−15.8%; 4.9%)	0.274
Gestinal age (weeks) ^c^	1.003	(0.982; 1.025)	0.3% (−1.8%; 2.5%)	0.769

The model was fitted as a GLM with a gamma distribution and a log-link function. Deviance-based pseudo-R^2^ = 0.271. Nagelkerke’s R^2^ = 0.271. ^a^ The cumulative calcifediol dose was calculated as the daily dose [µg/kg/day] multiplied by the number of days between measurements. ^b^ The cumulative calcifediol dose variable was transformed as log(1 + cumulative dose). ^c^ Gestational age was centered at 40 weeks of gestation.

## Data Availability

The original contributions presented in the study are included in the article; further inquiries can be directed to the corresponding author.

## References

[1] Degrassi I., Leonardi I., Di Profio E., Montanari C., Zuccotti G., Verduci E. (2023). Fat-soluble vitamins deficiency in pediatric cholestasis: A scoping review. Nutrients.

[2] Chongthavornvasana S., Lertudomphonwanit C., Mahachoklertwattana P., Korwutthikulrangsri M. (2023). Determination of optimal vitamin D dosage in children with cholestasis. BMC Pediatr..

[3] Fawaz R., Baumann U., Ekong U., Fischler B., Hadzic N., Mack C.L., McLin V.A., Molleston J.P., Neimark E., Ng V.L. (2017). Guideline for the evaluation of cholestatic jaundice in infants: Joint recommendations of the North American Society for Pediatric Gastroenterology, Hepatology, and Nutrition and the European Society for Pediatric Gastroenterology, Hepatology, and Nutrition. J. Pediatr. Gastroenterol. Nutr..

[4] Suchy F.J. (2004). Neonatal cholestasis. Pediatr. Rev..

[5] Giustina A., Bilezikian J.P., Adler R.A., Banfi G., Bikle D.D., Binkley N.C., Bollerslev J., Bouillon R., Brandi M.L., Casanueva F.F. (2024). Consensus statement on vitamin D status assessment and supplementation: Whys, whens, and hows. Endocr. Rev..

[6] Venkat V.L., Shneider B.L., Magee J.C., Turmelle Y., Arnon R., Bezerra J.A., Hertel P.M., Karpen S.J., Kerkar N., Loomes K.M. (2014). Total serum bilirubin predicts fat-soluble vitamin deficiency better than serum bile acids in infants with biliary atresia. J. Pediatr. Gastroenterol. Nutr..

[7] Kim Y.J., Lim G., Lee R., Chung S., Son J.S., Park H.W. (2023). Association between vitamin D level and respiratory distress syndrome: A systematic review and meta-analysis. PLoS ONE.

[8] Treiber M., Mujezinović F., Pečovnik Balon B., Gorenjak M., Maver U., Dovnik A. (2020). Association between umbilical cord vitamin D levels and adverse neonatal outcomes. J. Int. Med. Res..

[9] Wang S., Wang M., Yu X., Cao C., Ding Y., Lv M., Liu Y., Chu M., Fang K., Liao Z. (2025). Nonlinear relationship between vitamin D status on admission and bronchopulmonary dysplasia in preterm infants. Pediatr. Res..

[10] Doğan P., Özkan H., Köksal Fatma N., Çelebi S., Bağcı O., Topçu M., Güney Varal İ. (2021). The role of low 25-hydroxyvitamin D levels in preterm infants with late-onset sepsis. Fetal Pediatr. Pathol..

[11] Yin X., Xu S., Zhang X., Li L., Xi H., Ma L., Sun M., Yang P., Li X., Jiang H. (2024). The association between serum 25-hydroxyvitamin D levels and retinopathy of prematurity in preterm infants. Front. Pediatr..

[12] Song F., Li W., Chen Z., Maimaijiang A., Wang Y. (2025). 25OH vitamin D3 in biliary atresia: A simple and under-utilised diagnostic method. Front. Pediatr..

[13] Ukarapong S., Zegarra W., Navarrete C., Seeherunvong T., Berkovitz G. (2019). Vitamin D status among preterm infants with cholestasis and metabolic bone disease. Pediatr. Res..

[14] Kołodziejczyk-Nowotarska A., Bokiniec R., Seliga-Siwecka J. (2021). Monitored supplementation of vitamin D in preterm infants: A randomized controlled trial. Nutrients.

[15] Natarajan C.K., Sankar M.J., Agarwal R., Pratap O.T., Jain V., Gupta N., Gupta A.K., Deorari A.K., Paul V.K., Sreenivas V. (2014). Trial of daily vitamin D supplementation in preterm infants. Pediatrics.

[16] Shneider B.L., Magee J.C., Bezerra J.A., Haber B., Karpen S.J., Raghunathan T., Rosenthal P., Schwarz K., Suchy F.J., Kerkar N. (2012). Efficacy of fat-soluble vitamin supplementation in infants with biliary atresia. Pediatrics.

[17] Kamath B.M., Alonso E.M., Heubi J.E., Karpen S.J., Sundaram S.S., Shneider B.L., Sokol R.J. (2022). Fat-soluble vitamin assessment and supplementation in cholestasis. Clin. Liver Dis..

[18] Płudowski P., Kos-Kudła B., Walczak M., Fal A., Zozulińska-Ziółkiewicz D., Sieroszewski P., Peregud-Pogorzelski J., Lauterbach R., Targowski T., Lewiński A. (2023). Guidelines for preventing and treating vitamin D deficiency: A 2023 update in Poland. Nutrients.

[19] Pawłowska J., Socha P. (2016). Suplementacja składnikami odżywczymi u dzieci z przewlekłą cholestazą. Stand. Med. Pediatr..

[20] Baker A., Stevenson R., Dhawan A., Goncalves I., Socha P., Sokal E. (2007). Guidelines for nutritional care for infants with cholestatic liver disease before liver transplantation. Pediatr. Transplant..

[21] Mouzaki M., Bronsky J., Gupte G., Hojsak I., Jahnel J., Pai N., Quiros-Tejeira R.E., Wieman R., Sundaram S. (2019). Nutrition support of children with chronic liver diseases: A joint position paper of the North American Society for Pediatric Gastroenterology, Hepatology, and Nutrition and the European Society for Pediatric Gastroenterology, Hepatology, and Nutrition. J. Pediatr. Gastroenterol. Nutr..

[22] Shen Y.-M., Wu J.-F., Hsu H.-Y., Ni Y.-H., Chang M.-H., Liu Y.-W., Lai H.-S., Hsu W.-M., Weng H.-L., Chen H.-L. (2012). Oral absorbable fat-soluble vitamin formulation in pediatric patients with cholestasis. J. Pediatr. Gastroenterol. Nutr..

[23] Mancell S., Islam M., Dhawan A., Whelan K. (2022). Fat-soluble vitamin assessment, deficiency and supplementation in infants with cholestasis. J. Hum. Nutr. Diet..

[24] Dong R., Sun S., Liu X.-Z., Shen Z., Chen G., Zheng S. (2017). Fat-soluble vitamin deficiency in pediatric patients with biliary atresia. Gastroenterol. Res. Pract..

[25] Zhuang P., Sun S., Dong R., Chen G., Huang Y., Zheng S. (2019). Associations between vitamin D and liver function and liver fibrosis in patients with biliary atresia. Gastroenterol. Res. Pract..

[26] Ng J., Paul A., Wright N., Hadzic N., Davenport M. (2016). Vitamin D levels in infants with biliary atresia: Pre- and post-Kasai portoenterostomy. J. Pediatr. Gastroenterol. Nutr..

[27] Jodar E., Campusano C., de Jongh R.T., Holick M.F. (2023). Calcifediol: A review of its pharmacological characteristics and clinical use in correcting vitamin D deficiency. Eur. J. Nutr..

[28] Quesada-Gomez J.M., Bouillon R. (2018). Is calcifediol better than cholecalciferol for vitamin D supplementation?. Osteoporos. Int..

[29] Heubi J.E., Hollis B.W., Tsang R.C. (1990). Bone disease in chronic childhood cholestasis II. Better absorption of 25-OH vitamin D than vitamin D in extrahepatic biliary atresia. Pediatr. Res..

[30] Jensen M., Abu-El-Haija M., Bishop W., Rahhal R. (2015). Difficulty achieving vitamin D sufficiency with high-dose oral repletion therapy in infants with cholestasis. J. Pediatr. Gastroenterol. Nutr..

[31] da Rocha C.R.M., Guaragna-Filho G., Kieling C.O., Adami M.R., Guedes R.R., Vieira S.M.G. (2023). Daily vitamin D supplementation improves vitamin D deficiency in patients with chronic liver disease. J. Pediatr. Gastroenterol. Nutr..

[32] Gordon C.M., Williams A.L., Feldman H.A., May J., Sinclair L., Vasquez A., Cox J.E. (2008). Treatment of hypovitaminosis D in infants and toddlers. J. Clin. Endocrinol. Metab..

[33] Flores-Aldana M., Rivera-Pasquel M., García-Guerra A., Pérez-Cortés J.G., Bárcena-Echegollén J.E. (2023). Effect of vitamin D supplementation on 25(OH)D status in children 12–30 months of age: A randomized clinical trial. Nutrients.

[34] Pappa H.M., Mitchell P.D., Jiang H., Kassiff S., Filip-Dhima R., DiFabio D., Quinn N., Lawton R.C., Varvaris M., van Straaten S. (2012). Treatment of vitamin D insufficiency in children and adolescents with inflammatory bowel disease: A randomized clinical trial comparing three regimens. J. Clin. Endocrinol. Metab..

[35] Tripkovic L., Lambert H., Hart K., Smith C.P., Bucca G., Penson S., Chope G., Hyppönen E., Berry J., Vieth R. (2012). Comparison of vitamin D2 and vitamin D3 supplementation in raising serum 25-hydroxyvitamin D status: A systematic review and meta-analysis. Am. J. Clin. Nutr..

[36] Saron M.L.G., Godoy H.T., Hessel G. (2009). Nutritional status of patients with biliary atresia and autoimmune hepatitis related to serum levels of vitamins A, D and E. Arq. Gastroenterol..

[37] Tessitore M., Sorrentino E., Schiano Di Cola G., Colucci A., Vajro P., Mandato C. (2021). Malnutrition in pediatric chronic cholestatic disease: An up-to-date overview. Nutrients.

[38] Yang C.H., Perumpail B.J., Yoo E.R., Ahmed A., Kerner J.A. (2017). Nutritional needs and support for children with chronic liver disease. Nutrients.

[39] Samra N.M., Emad El Abrak S., El Dash H.H., El Said El Raziky M., El Sheikh M.A. (2018). Evaluation of vitamin D status bone mineral density and dental health in children with cholestasis. Clin. Res. Hepatol. Gastroenterol..

